# DMAHDM@MPC nanoparticles in orthodontic adhesive inhibit cariogenic bacteria and sugar metabolism to prevent enamel demineralization

**DOI:** 10.1016/j.mtbio.2025.101969

**Published:** 2025-06-09

**Authors:** Chengjun Su, Mengyao Zhu, Yiman Guo, Jiachen Sun, Miao Liu, Yansong Ma, Yan Xu, Yuxing Bai, Xiaoxia Che, Ning Zhang

**Affiliations:** a Department of Orthodontics, School of Stomatology, Capital Medical University, Beijing, 100070, China; b CAS Key Lab of Colloid, Interface and Chemical Thermodynamics, Institute of Chemistry, Chinese Academy of Sciences, Beijing, 100190, China

**Keywords:** Fixed orthodontic treatment, Modified orthodontic bonding agent, Antimicrobial properties, Clinical evaluation, Metagenomic sequencing

## Abstract

During orthodontic treatment, poor oral hygiene often facilitates the proliferation of cariogenic bacteria, particularly *Streptococcus mutans*, leading to lactic acid accumulation and subsequent enamel demineralization. To mitigate this issue, Dimethylaminohexadecyl methacrylate (DMAHDM) was incorporated onto the protein-repellent surface of 2-Methacryloyloxyethyl phosphorylcholine (MPC), resulting in the formation of a DMAHDM@MPC composite. This composite was then integrated into resin-modified glass ionomer cement (RMGIC) to develop an antimicrobial orthodontic adhesive, termed RMGIC + MPC + DMAHDM (RMD). This study demonstrated that DMAHDM@MPC nanoparticles self-assembled into a core-shell structure, thereby enhancing the antimicrobial activity. A six-month randomized controlled trial (RCT) involving 29 orthodontic patients, along with metagenomic and metabolomic analyses, revealed that RMD significantly reduced plaque accumulation by selectively inhibiting pathogenic bacteria while preserving beneficial microbiota. Additionally, MPC was shown to competitively bind to sucrose-6-phosphatase (SPP) in pathogenic bacteria, inhibiting sucrose synthesis and carbohydrate metabolism, thus reducing the production of organic acids. In conclusion, RMD effectively prevents enamel demineralization by selectively targeting cariogenic bacteria and their associated sugar metabolism pathways during orthodontic treatment.

## Highlights

• This study combined clinical trial, metagenomic sequencing, and multi-omics analyses.
• The bonding agent contains synthesized nanomaterials for antibacterial effects.
• The adhesive exerts antimicrobial effects by binding to sucrose-6-phosphatase.
• Blocking sugar metabolism in cariogenic bacteria prevents enamel demineralization.

## Graphical abstract

A schematic diagram illustrating the mechanism of a novel orthodontic adhesive based on RMD for inhibiting cariogenic bacteria and preventing enamel demineralization. (A) Schematic representation of RMD preparation. (B) A split-mouth design employed for the study. (C) Mechanism of RMD selectively targeting cariogenic bacteria, inhibiting sucrose synthesis and downstream carbohydrate metabolism, including organic acid production. (D) Prevention of enamel demineralization as a result of the intervention.

**Figure:**
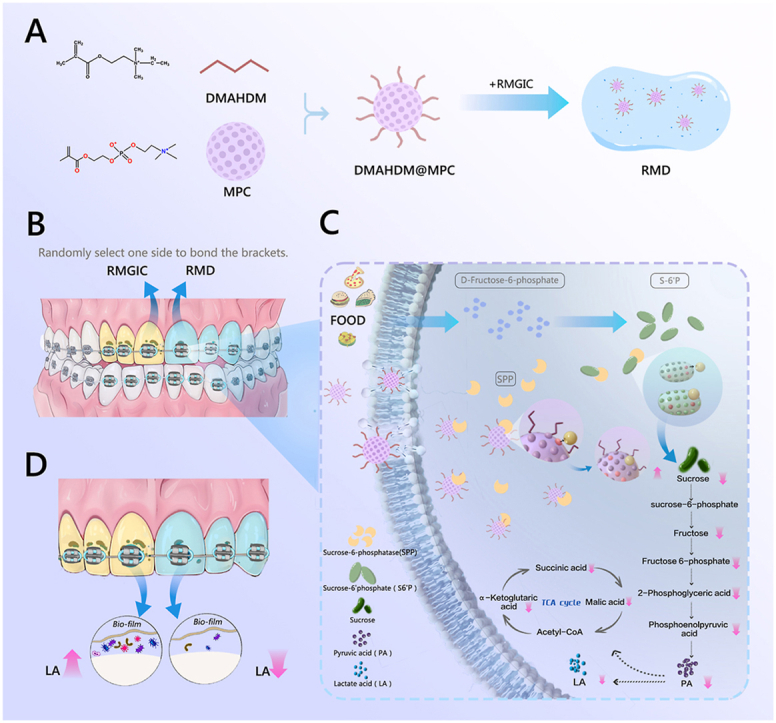

## 1 Introduction

The oral microbiome plays a fundamental role in systemic health through various pathways, including oral-cardiac [1], oral-brain [2], and oral-gut [3] axes. Orthodontic treatment, however, disrupts this ecosystem balance. The use of orthodontic appliances serves as both an external stressor and a foreign object, modifying patients' oral hygiene practices [4]. Studies highlight that Metallic brackets of fixed appliances enhance bacterial adhesion via irregular surfaces and increased surface roughness [5]. And the adhesives around the brackets could easily induce biofilm accumulation [6] due to its rough surfaces and the gap interface of adhesives and the tooth surface [7]. Initial colonization and adhesion drive biofilm formation through complex interactions between species [4]. Altered oral environments particularly promote the proliferation of *Streptococcus mutans* (*S. mutans*) and other cariogenic bacteria, which convert dietary carbohydrates into organic acids, leading to biofilm formation and enamel demineralization [8,9]. As a result, microbial imbalances increase the risk of white spot lesions and dental caries, while also fostering conditions that contribute to periodontal disease [10,11].

Compared to conventional clinical methods to effectively prevent these complications (e.g., mouth rinse, fluoride gel, varnish, and sealant) [12,13], antibacterial orthodontic adhesives offer a sustainable strategy to prevent enamel demineralization without requiring repeated applications, thereby minimizing reliance on patient compliance. Therefore, it is necessary to develop adhesives with antibacterial properties.

Conventional fluoride-releasing Resin-Modified Glass Ionomer Cement (RMGIC) has been used as a preventive measure, but its efficacy diminishes over time as fluoride release decreases [14]. To address this limitation, nanomaterials have gained attention due to their ability to enhance polymeric composites through the incorporation of nano-sized particles and their potential for caries prevention [15,16], with both inorganic and organic nanoparticles being investigated for these purposes. Inorganic nanoparticles, such as ZnO NPs, TiO_2_ NPs, CuO NPs, and nano-fluorapatite, have demonstrated significant antibacterial properties when integrated into RMGIC [17,18]. However, their potential cytotoxicity limits broader use and may negatively affect the mechanical properties of RMGIC [19,20].

Organic antimicrobial materials, particularly quaternary ammonium compounds (QACs) such as dimethylaminohexadecyl methacrylate (DMAHDM), have attracted attention due to their biocompatibility and aesthetic properties. DMAHDM disrupts bacterial membranes via its 16-carbon alkyl chain, leading to bacterial cell death [21]. Incorporating DMAHDM into RMGIC has been shown to significantly enhance antibacterial efficacy while preserving mechanical properties [22]. However, protein adsorption from saliva reduces its antimicrobial effectiveness [23]. To overcome this issue, 2-methacryloyloxyethyl phosphorylcholine (MPC), a highly hydrophilic, biocompatible compound with protein-repellent properties, has been integrated into biomedical materials to inhibit bacterial adhesion [24,25].

When combined with DMAHDM and incorporated into RMGIC, MPC enhances the material's antimicrobial properties, strengthening its antibacterial activity while maintaining biocompatibility [26]. This combination, in the form of RMGIC containing DMAHDM and MPC (RMD), has also shown significant antibacterial effects in an initial clinical trial [5]. However, this study was limited by small sample sizes, potentially introducing bias, and the use of 16S rRNA sequencing, which only identifies bacteria at the genus level, hindering species-level identification and functional annotation of the microbiome. Further optimization of DMAHDM and MPC assembly is needed, and the antimicrobial mechanisms of RMD in high-sugar oral environments remain poorly understood.

To address these challenges, this study aims to: (1) elucidate the microscopic structure and assembly patterns between MPC and DMAHDM components, (2) investigate their synergistic antimicrobial mechanism and molecular-level influence on dental biofilm formation, and (3) evaluate their potential in preventing enamel demineralization.

## 2 Materials and methods

### 2.1 Ethics statement

The study was approved by Ethics Committee of Beijing Stomatological Hospital Affiliated with Capital Medical University (IRB No. CMUSH-IRB-KJ-PJ-2023-59). All participants were informed about the study's objectives and provided written informed consent for voluntary participation.

### 2.2 Preparation of DMAHDM

The synthesis of DMAHDM [27] was conducted via a modified Menschutkin reaction, utilizing 2-(dimethylamino) ethyl methacrylate (DMAEMA, Tokyo Chemical Industry, Japan) as the tertiary amine substrate and 1-bromohexadecane (BHD, Monomer-Polymer and Dajac Labs, USA) as the organic halide. Specifically, 10 mM DMAEMA, 10 mM BHD, and 3.0 g ethanol were added to a 20 mL conical flask. The reaction system was magnetically stirred at 70 °C for 24 h. After complete reaction, the ethanol solvent was evaporated, ultimately yielding a clear, colorless DMAHDM monomer.

### 2.3 Preparation of DMAHDM@MPC

Equal masses of DMAHDM and MPC (730114, Sigma-Aldrich, USA) were separately dissolved in ethanol. DMAHDM was fully dissolved and subsequently combined with the MPC solution. The resulting mixture was incubated in the dark on an orbital shaker at room temperature for 1 h. The resulting mixture was then freeze-dried using a lyophilizer in preparation for subsequent characterization.

### 2.4 Characterization of DMAHDM@MPC

The particle size and surface charge of MPC, DMAHDM, and DMAHDM@MPC were measured using Dynamic Light Scattering (DLS). Appropriate amounts of each sample were dissolved in water and analyzed using a Zetasizer Nano ZS90 (Malvern Instruments, Malvern, UK), with five measurements conducted per sample. The morphology of MPC, DMAHDM, and DMAHDM@MPC was analyzed using Transmission Electron Microscopy (TEM). Sample solutions were deposited onto copper grids and allowed to air-dry. TEM observations were carried out using a JEM-1011 (JEOL, Japan) at an accelerating voltage of 100 kV, with five measurements per sample. Additionally, the molecular structure and composition of MPC, DMAHDM, and DMAHDM@MPC were determined using UV–Vis spectroscopy (U-3010, HITACHI, Japan) and Fourier-transform infrared (FTIR) spectroscopy (VERTEX 70v, Bruker, Germany), with five measurements for each sample. The surface chemical composition of DMAHDM@MPC was analyzed using X-ray Photoelectron Spectroscopy (XPS). Powder samples were secured between two pieces of aluminum foil with double-sided tape, pressed, and cut into 0.3 mm × 0.3 mm sections for testing. Spectra were recorded using an ESCALAB 250XI (Thermo Fisher Scientific, USA), with five measurements taken per sample to ensure accuracy and reproducibility.

### 2.5 Preparation of RMD and RMGIC

6 % freeze-dried DMAHDM@MPC powder by weight was added to the RMGIC powder (Fuji ORTHO LC, GC, Japan) and thoroughly mixed. According to the material instructions, the recommended powder-to-liquid mass ratio for mixing is 2.5:1. The resulting powder mixture was then combined with RMGIC liquid at a weight ratio of 2.5:1 to synthesize RMD. The material has been inspected by the National Institutes for Food and Drug Control (NIFDC) in China and certified for its biological safety (IRB No. QH202200803).

In parallel, RMGIC powder was blended with RMGIC liquid at the same mass ratio of 2.5:1 to prepare RMGIC.

### 2.6 Bacterial growth curves analysis

To evaluate the impact of RMD on the growth curves of human oral multispecies biofilm bacteria, RMD, RMGIC, and Transbond XT adhesive Control (3M Unitek, Monrovia, California, USA) specimens were prepared. The lids of 48-well plates were used as molds, with the materials evenly spread across the surface. A medical polyester film (9901P, 3M, USA) was placed over the materials, and light pressure was applied to ensure a smooth, uniform surface. The samples were light-cured for 40 s, yielding circular disc specimens with a diameter of 10 mm and a thickness of 2 mm. Next, human oral multispecies biofilms were cultured according to the method described by Zhang et al. [28]. Five healthy adult volunteers were selected as saliva donors, with the condition that none had taken antibiotics in the past three months. Donors refrained from brushing their teeth for 24 h and fasted for at least 2 h before unstimulated saliva donation. Equal volumes of unstimulated saliva from each donor were pooled and diluted 1:50 in McBain medium containing 0.5 % sucrose. 1.5 mL of the inoculated medium was added to 24-well culture plates, and the samples were incubated at 37 °C in a 5 % CO_2_ atmosphere for 8 h. The biofilm was transferred to a fresh plate with new McBain medium and cultured for an additional 16 h. This process was repeated once more, with biofilm culturing continuing for another 24 h to allow for the formation of a mature human oral multispecies biofilm. Finally, the RMD, RMGIC and Transbond XT control (referred to as TB Ctrl) specimens were sterilized using ethylene oxide. Three time groups were established and co-cultured with biofilm for one, two, and three days, respectively, with distinct specimens used for each time group to ensure data independence. At the end of days 1, 2, and 3, the corresponding specimens were removed from the culture medium in the wells. The specimens were gently rinsed three times with PBS to remove non-adherent bacteria. The rinsed specimens were then immersed in centrifuge tubes containing 1.5 mL of PBS and subjected to ultrasonic treatment (40 kHz, 5 min) to disperse the biofilm. After vortex-mixing thoroughly, 1 mL of the suspension was collected, and the optical density (OD) at 600 nm was measured to monitor bacterial growth. Growth curves were plotted based on the collected data.

### 2.7 Characterization of biofilm

The RMD and RMGIC specimens were sterilized with ethylene oxide and co-cultured with human oral multispecies biofilms for 2 days to facilitate biofilm formation on the specimen surfaces. At an OD of 0.01 (600 nm), biofilm formation was clearly evident. After rinsing with phosphate-buffered saline (PBS) to remove unattached bacteria, the specimens were fixed in 2.5 % glutaraldehyde for 4 h, followed by dehydration with graded ethanol. The specimens were then sputter-coated with platinum. Finally, the samples were analyzed using a scanning electron microscope (SEM) (S-4800, HITACHI, Japan).

### 2.8 Clinical study design

A superiority randomized controlled trial (RCT) was conducted to assess the effects of RMD on plaque biofilm formation around orthodontic brackets during treatment. Participants were selected according to stringent inclusion criteria: a full set of permanent teeth, intact enamel, no history of caries, periodontal disease, previous orthodontic treatment, extractions, or surgery. All participants were non-smokers, free from systemic diseases, and had not taken long-term medications or antibiotics in the past three months. Written informed consent was obtained from each participant. The sample size was estimated based on the incidence of enamel demineralization on maxillary anterior teeth and the expected reduction in demineralization due to the intervention. 40 participants were initially recruited. After accounting for attrition and the exclusion of non-qualifying samples, 29 orthodontic patients (18 females, 11 males; average age 14.2 ± 2.1 years) were ultimately enrolled.

A split-mouth design, as described by Lesaffre et al. [29] was employed to minimize individual variation in oral microbiome profiles. For each participant, three teeth in one maxillary quadrant (central incisor, lateral incisor, and canine) were bonded with RMD, while the corresponding teeth in the opposite quadrant were bonded with commercial RMGIC as controls. All bracket bonding procedures were performed by the same operator.

### 2.9 Cement preparation and bracket bonding

Prior to bonding, the teeth were cleaned, etched with 37 % phosphoric acid for 30 s, air-dried, and subsequently coated with either RMD or RMGIC. A 3.0 N force was applied for 5 s to ensure uniform cement thickness, followed by 40 s of light curing. All bonding procedures were performed by the same operator. Participants were instructed to use fluoride-free toothpaste and avoid products that could alter the oral microbiome.

### 2.10 Clinical evaluation

To assess the influence of RMD and RMGIC on enamel demineralization and gingivitis development, relevant clinical indices were evaluated at four time points: one week before bracket bonding (T0), and at one (T1), three (T2), and six months (T3) post-bonding. Enamel demineralization around the brackets was evaluated visually using the enamel decalcification index (EDI), with the EDI score calculated based on Banks et al. [30]. The mesial, distal, occlusal, and gingival areas surrounding six teeth were scored for decalcification, and the EDI score for each participant was determined by dividing the total score by the number of quadrants assessed. To further quantify enamel demineralization severity, the DIAGNOdent pen 2190 (Kavo, Biberach, Germany), a laser fluorescence device, was employed, following the same scoring method as the EDI [31]. Measurements were taken using a cylinder sapphire tip designed for occlusal surfaces, positioned 1 mm away from the bracket in each quadrant zone, with three replicate readings per zone. The DIAGNOdent (Dd) score for each tooth was calculated as the average of the four quadrant readings, and the individual score was the average of all bonded teeth. In parallel with these assessments of enamel demineralization, plaque accumulation in the gingival area was evaluated. At each time point, before performing oral hygiene and collecting plaque samples, plaque accumulation was assessed using the Turesky modification of the Quigley and Hein plaque index (Turesky QH PI) [32]. Additionally, gingival index (GI) measurements related to gingivitis development were performed using a periodontal probe according to standard procedures [33].

### 2.11 Supragingival plaque collection

Supragingival plaque samples were collected at T0 and T3. To ensure accuracy, participants were instructed to refrain from any oral hygiene procedures for 12 h before sampling and to rinse with warm water, followed by air drying for 30 s prior to collection. A single trained clinician used sterile swabs (FLOQSwabs, 501CS01, Copan) to scrape plaque from three teeth bonded with the same material around the brackets, pooling the plaque into one sample. Each participant provided two plaque samples at each time point: one from RMGIC-bonded teeth and one from RMD-bonded teeth. The collected swabs (3–5 per tube) were cut short, placed into sterile Eppendorf tubes, flash-frozen in liquid nitrogen, and stored at −80 °C. Samples were then shipped on dry ice for further analysis.

### 2.12 Metagenomic analysis

DNA was extracted from plaque samples using the CTAB method, and its quality was assessed with an Agilent 2100 Bioanalyzer. Qualified samples were stored at −80 °C for further processing and subsequently sent to Wekemo Tech Co., Ltd. (Shenzhen, China) [34] DNA libraries were constructed using the NEB Next® Ultra™ Kit (NEB, USA, Catalog #: E7370L), amplified by Real-time quantitative PCR (RT-qPCR), and purified with the AMPure XP system (Beverly, USA). The quality and concentration of the libraries were evaluated using the Agilent 5400 system (Agilent, USA) and quantified by RT-qPCR (1.5 nM). Sequencing was performed on the Illumina NovaSeq platform. The data were processed using Trimmomatic [35], Bowtie2 [36], Kraken2, and Bracken [37] to remove low-quality reads and identify species composition. Functional gene analysis was done with DIAMOND (version 0.7.10.59). Differential gene expression was analyzed using DESeq2, with p-values adjusted for multiple testing using the Benjamini-Hochberg (BH) method. Significant genes were defined as having |log2(FoldChange)| > 1 and adjusted p-values (*P*.adj) < 0.05. The Kyoto Encyclopedia of Genes and Genomes (KEGG) pathway enrichment analysis was performed using the R package clusterProfiler, mapping gene IDs to the KEGG Orthology (KO) system to identify significantly enriched pathways associated with differentially expressed genes [38]. The species origin of functional genes was further determined using the HUMAnN3 tool to provide deeper insights into the biological processes involved.

### 2.13 Real-time quantitative PCR

Based on the metagenomic sequencing results, the quantification of *S. mutans*, *Selenomonas sputigena* (*S. sputigena*), *Rothia aeria* (*R. aeria*) and *Rothia mucilaginosa* (*R. mucilaginosa*) in the plaques of clinical specimens was further carried out and validated using RT-qPCR. DNA was extracted with QIAamp DNA Mini Kit (Qiagen, USA). In addition, gene expression levels of SPP in *S. mutans* when co-cultured with RMD and RMGIC were assessed. The strain was initially cultured on MSA solid medium under anaerobic conditions (80 % N_2_, 10 % H_2_, 10 % CO_2_) at 37 °C. After ensuring purity through subculturing, the strain was transferred to 10 mL of BHI liquid medium and cultured under the same anaerobic conditions for 24–48 h. Total RNA was extracted using the RNA simple Total RNA Kit (Tiangen, DP419, China) according to the manufacturer's instructions. DNA and RNA quality and quantity were assessed using a Nanodrop 2000 spectrophotometer (Thermo Scientific, Waltham, MA, USA) and confirmed the integrity on 1 % agarose gels. RT-qPCR was performed using the Premix Taq RT-qPCR System (Takara Bio, Kyoto, Japan). The primers for bacterial quantification and SPP expression level detection were selected based on relevant literature (Table 1) [[39], [40], [41]]. RT-qPCR conditions were as follows: initial denaturation at 94 °C for 3 min; 40 cycles of denaturation at 94 °C for 5 s, annealing at 59 °C/58 °C for 15 s, and extension at 72 °C for 10 s. The universal gene was utilized as the housekeeping gene, and all samples were analyzed in triplicate.

**Table 1:** The primers used for bacterial quantification and SPP.

Primer name	Primer sequence (5′-3′)
Universal F	TCCTACGGGAGGCAGCAGT
Universal R	GGACTACCAGGGTATCTAATCCTGTT
*S. mutans* F	GCCTACAGCTCAGAGATGCTATTCT
*S. mutans* R	GCCATACACCACTCATGAATTGA
*Selenomonas sputigena* F	AGAGTTTGATCCTGGCTCAG
*Selenomonas sputigena* R	TCAATATTCTCAAGCTCGGTT
*Rothia aeria* F	GTGCTTGCACGTGGATTAGTGG
*Rothia aeria* R	TGACGCGATCTAATGCATGTCAAG
*Rothia mucilaginosa* F	GCCTAGCTTGCTAGGTGGAT
*Rothia mucilaginosa* R	GCAGGTACCGTCAATCTCTC
*sucrose-6-phosphatase* F	TCCCGATGGCCGTGTTTATG
*sucrose-6-phosphatase* R	TTGACGAGGCTCAAAGCTCC

### 2.14 Measurement of sucrose content in metabolites

*S. mutans* was initially cultured on MSA solid medium under anaerobic conditions (80 % N_2_, 10 % H_2_, 10 % CO_2_) at 37 °C. After ensuring purity through subculturing, the strain was transferred to 10 mL of BHI liquid medium and cultured under the same anaerobic conditions. Cultures of *S. mutans* co-cultured with RMD and RMGIC have been prepared, and their OD at 600 nm was measured to be 0.01. The bacterial cultures were transferred to clean Eppendorf tubes and centrifuged at 25 °C, 4000 g for 10 min. The supernatant was collected, and sucrose content in the RMD and RMGIC groups was measured using the Sucrose Assay Kit BC2460 (Solarbio, Beijing, China) according to the manufacturer's instructions, with distilled water used as the blank control. The absorbance values were determined at 480 nm using a visible spectrophotometer.

### 2.15 Molecular docking and visualization

Protein structures were obtained from the PDB database (https://www.rcsb.org/) and small molecule data from the PubChem database (https://pubchem.ncbi.nlm.nih.gov/). Small molecules were processed using Open Babel software. Molecular docking between proteins and small molecules was performed using AutoDock 4.2 and AutoDockTools 1.5.7. The interactions from molecular docking were visualized using PyMOL software, generating structural models of the binding modes between the protein and small molecule ligands. Sequence alignment was subsequently performed using MEGA 7, and the amino acid sequences of SPP across different oral bacterial species were visualized using ESPript 3.0.

### 2.16 Sample preparation and LC-MS/MS analysis for metabolomics

Based on the metagenomic sequencing results, metabolomic analysis was further employed to evaluate the metabolic products and microbial functions of *S. mutans* under co-culture conditions with RMD and RMGIC. Cultures of *S. mutans* were prepared in a sucrose-free medium, co-cultured with RMD and RMGIC to a final OD value of 0.01. After washing the bacterial cultures three times with PBS, the samples were centrifuged at 12,000×*g* for 10 min at 4 °C, and the supernatants were aliquoted and stored at −80 °C. For metabolite extraction, 100 μL of each bacterial culture was mixed with 1200 μL of methanol, vortexed, and centrifuged at 13,000 rpm for 10 min. The supernatant was removed after centrifugation, and the remaining pellet was stored for further analysis. Metabolite separation was performed using an Ultra 3000 UHPLC system with an ACQUITY UPLC® HSS T3 column, with a gradient elution of 0.1 % formic acid in water (A) and 0.1 % formic acid in acetonitrile (B). Mass spectrometry was conducted on a Thermo Q Exactive Plus equipped with an electrospray ionization source, in both positive and negative ion modes. The data were analyzed using MarkerLynx software for peak extraction, alignment, and normalization, followed by molecular formula prediction and database matching for relative quantification.

### 2.17 Statistical analysis

Statistical analysis was performed using SPSS 25.0 and R software (4.4.1). First, normality tests were conducted for continuous data using the Shapiro-Wilk test. Data conforming to normal distribution were expressed as mean ± standard deviation ($\overset{®}{x}$ ± s), while non-normally distributed data were expressed using quartiles. Homogeneity of variance was tested with an F-test; data with homogeneous variances were analyzed using t-tests or one-way analysis of variance (ANOVA), while data with heterogeneous variances were analyzed using the rank-sum test. Categorical data were expressed as counts (percentages) and analyzed using the chi-square test or Fisher's exact test. A bilateral *P* value of <0.05 was considered statistically significant. Metabolomics data analysis was conducted using R, Python, and CentOS, with significant metabolites identified through principal component analysis (PCA) and orthogonal partial least squares discriminant analysis (OPLS-DA), applying a *P*.adj <0.01 and an absolute log2FC ≥ 1.

## 3 Results

### 3.1 Preparation and characterization of DMAHDM@MPC

**Fig. 1:**
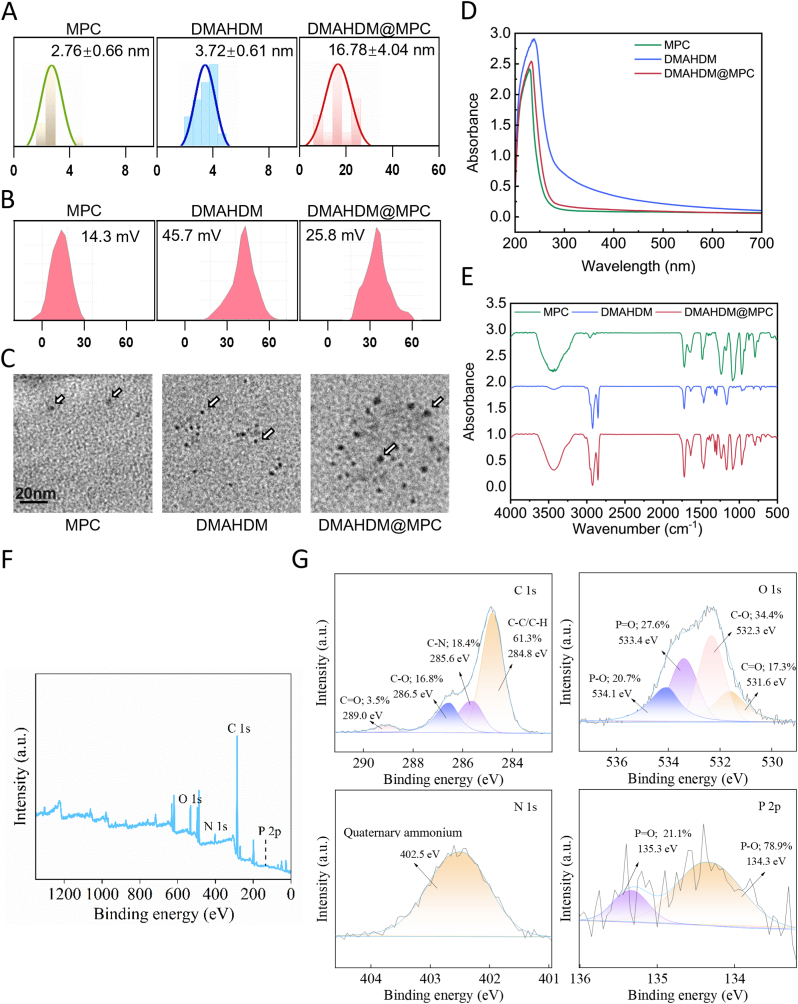
Characterization of DMAHDM@MPC. (**A**) Particle size analysis of MPC, DMAHDM, and DMAHDM@MPC. (**B**) Zeta potential analysis of MPC, DMAHDM, and DMAHDM@MPC. (**C**) TEM images of MPC, DMAHDM, DMAHDM@MPC. (**D**) The UV–Vis absorption spectra of MPC, DMAHDM, and DMAHDM@MPC. (**E**) Fourier transform infrared analysis of MPC, DMAHDM, and DMAHDM@MPC. (**F**) Full XPS spectra and (**G**) high-resolution XPS spectrum of DMAHDM@MPC.

The particle size distribution of DMAHDM@MPC was characterized using particle size analysis. As shown in Fig. 1A, MPC particles had an average size of 2.76 nm, while DMAHDM particles averaged 3.72 nm. Upon assembly, the average size of DMAHDM@MPC increased significantly to 16.78 nm. Zeta potential measurements revealed that DMAHDM@MPC had a zeta potential of 25.8 mV, compared to 14.3 mV for MPC and 45.7 mV for DMAHDM (Fig. 1B), highlighting changes in surface charge properties during assembly. TEM images (Fig. 1C) showed that MPC particles were approximately 2 nm in size, highly dispersed, and uniformly distributed without noticeable aggregation, indicating stable colloidal behavior. DMAHDM particles, about 3 nm in size, displayed irregular shapes and pronounced aggregation, suggesting that DMAHDM's amphiphilic nature and high surface activity promote the formation of clustered structures due to strong electrostatic interactions. In contrast, the DMAHDM@MPC assembly, approximately 16 nm in size, exhibited a more uniform and compact distribution, with a core-shell structure.

The UV–Vis absorption spectra (Fig. 1D) showed that MPC, DMAHDM@MPC, and DMAHDM exhibited distinct absorption peaks at approximately 233 nm, 235 nm, and 240 nm, respectively. The FTIR analysis (Fig. 1E) displayed that, DMAHDM@MPC had absorption peaks at 3300-3500 cm^−1^ (hydrogen bonds), 2900 cm^−1^ (C-H stretching), 1700 cm^−1^ (C=O stretching), 1600 cm^−1^ (C=N stretching), and 1000-1200 cm^−1^ (C-O-C stretching). These results confirm that the assembly of DMAHDM@MPC did not introduce new functional groups or chemical bonds, and the absorption features corresponded to the original chemical bonds of MPC and DMAHDM, indicating no chemical reaction during assembly.

XPS analysis of DMAHDM@MPC demonstrated the presence of the characteristic elemental peak (Fig. 1F). The high-resolution C 1s XPS spectrum identified four peaks corresponding to C-C (284.8 eV), C-N (285.6 eV), C-O (286.5 eV), and O-C-O (289.0 eV) bonds. Similarly, the O 1s spectrum revealed peaks corresponding to O-C-O (531.6 eV), C-O (532.3 eV), O-P-O (533.4 eV), and P-O (534.1 eV) bonds. The N 1s spectrum displayed a peak at 402.5 eV, consistent with the spectra of quaternary ammonium while the P 2p peaks at 134.3 eV (P-O) and 135.3 eV (O-P-O) matched the characteristic peaks of MPC (Fig. 1G).

Elemental composition analysis showed that the atomic percentage of P 2p in DMAHDM@MPC was 0.36 %, while N 1s was 3.96 %. Given that all nitrogen content originated from DMAHDM, the N/P ratio calculation suggested a molar ratio of approximately 10:1 between DMAHDM and MPC in the assembly. This ratio, along with the increased particle size and a core-shell structure observed in TEM images, confirms the orderly assembly of DMAHDM around the MPC core.

### 3.2 In vitro antibacterial efficacy of RMD

**Fig. 2:**
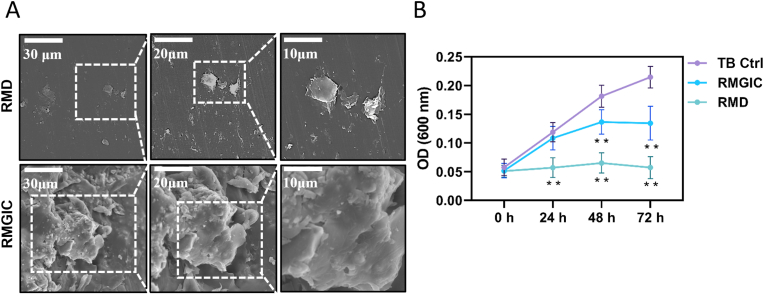
In vitro antibacterial efficacy of RMD. (**A**) SEM images of RMD and RMGIC co-cultured with full bacterial biofilms. (**B**)The bacterial growth curves of the Transbond XT control (referred to as TB Ctrl), RMGIC, and RMD groups. N = 5. All the results are presented as the mean ± SD. Statistical significance was determined using ANOVA followed by Tukey's multiple comparison test. ∗∗*P* < 0.01, versus the TB Ctrl group in the same time point.

Fig. 2A presents the SEM images of RMD and RMGIC co-cultured with full bacterial biofilms. RMGIC group displayed numerous irregular bacterial clusters densely packed on the surface, with a rough texture indicative of a higher propensity for biofilm formation on RMGIC. Conversely, RMD group exhibited a markedly reduced bacterial density, and the bacteria on the surface appeared deformed and shriveled, which are typical signs of bacterial inhibition or response to antimicrobial treatment.

Fig. 2B illustrates the bacterial growth curves of the Transbond XT control (referred to as TB Ctrl), RMGIC, and RMD groups. The TB Ctrl group exhibited continuous bacterial growth, peaking at 72 h. In the RMGIC group, although OD values initially increased, they stabilized after 48 h and remained consistently lower than those of the TB Ctrl group (*P* < 0.01), indicating a certain degree of antibacterial efficacy. In the RMD group, OD values initially increased slightly but rapidly stabilized at a much lower level after 24 h than in the TB Ctrl group (*P* < 0.01), demonstrating a significantly stronger antibacterial effect and effectively suppressing bacterial proliferation.

### 3.3 Clinical evaluation

**Fig. 3:**
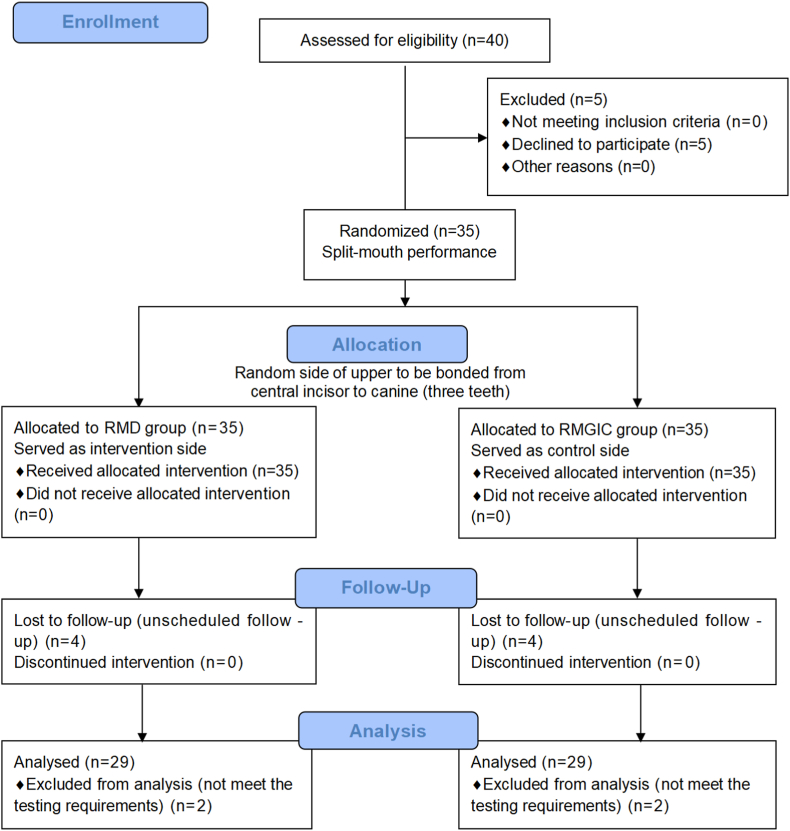
CONSORT Flow Diagram of the superiority randomized controlled trial (RCT).

35 patients agreed to enroll in the study (Fig. 3). Each participant in this study was performed by a split-mouth design. One random maxillary quadrant of each participant was assigned to RMD group, and the opposite quadrant was assigned to RMGIC control group. During the follow-up period, four participants were excluded from the study due to missed follow-up appointments and failure to attend the scheduled testing sessions. During the analysis phase, two participants' samples failed to meet the testing requirements and were therefore excluded. Finally, a total of 29 participants (18 females and 11 males) with a mean age of 14.2 ± 2.1 years were enrolled in the study. The RMD and RMGIC groups each included 87 upper anterior teeth (174 total), all derived from 29 patients, for analysis.

**Fig. 4:**
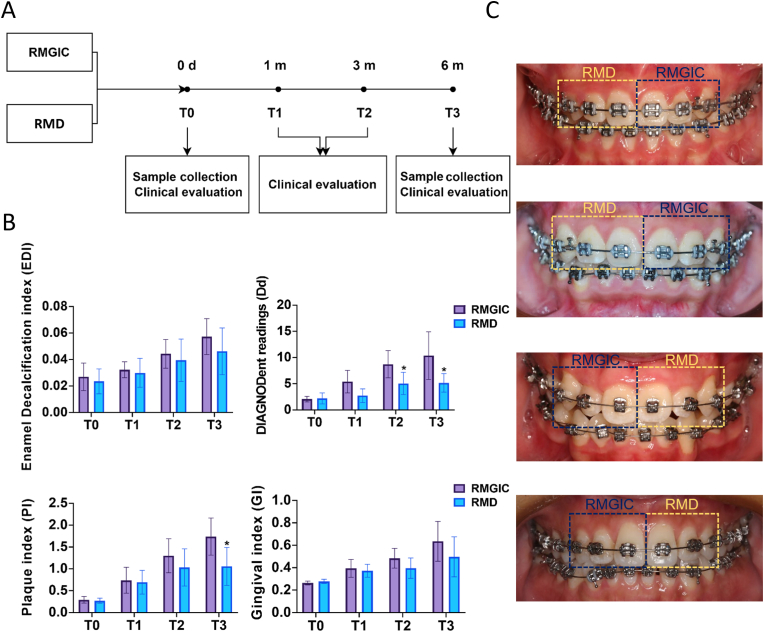
Clinical evaluation. (**A**) Study design for clinical evaluation and sample collection of RMGIC and RMD materials. Clinical evaluations and sample collections for both RMGIC and RMD groups were conducted at four key time points: T0 (0 days), T1 (one-month post-bonding), T2 (three months post-bonding) and T3 (six months post-bonding). (**B**) Clinical parameters at all time points, including enamel decalcification index (EDI), DIAGNODent readings (Dd), plaque index (PI), and gingival index (GI). Bar plots were presented as the mean with standard error. N = 87. All the results are presented as the mean ± SD. Statistical significance was determined using student *t*-test. ∗*P* < 0.05, versus the RMGIC group. (**C**) Intraoral photos of four patients at the T3 stage.

Clinical assessments were conducted at T0, T1, T2, and T3, as shown in Fig. 4A. During the six-month orthodontic treatment, enamel demineralization was assessed using the EDI and Dd readings, while periodontal health was evaluated through the PI and GI scores (Fig. 4B). The EDI scores showed an upward trend in both groups from T0 to T3, with the RMD group showing lower scores than the RMGIC group from T1 to T3. The Dd scores remained below the diagnostic threshold for demineralization (below 23) [42] in both groups from T0 to T3; however, the RMGIC group had significantly higher Dd scores than the RMD group at T2 and T3 (*P* < 0.05). The PI scores revealed significantly slower plaque accumulation in the RMD group at T3 compared to the RMGIC group (*P* < 0.05). Additionally, The GI scores also increased in both groups from T0 to T3, with the RMD group had lower scores than the RMGIC group from T1 to T3.

Furthermore, intraoral photographs of four patients at the T3 stage revealed that, consistent with clinical evaluations, the RMD side exhibited less enamel demineralization, gingival swelling, and bleeding around the brackets, with lower plaque accumulation compared to the RMGIC side (Fig. 4C).

Then, the debonding rates of orthodontic brackets bonded with RMGIC and RMD were evaluated at various time points. At T1, T2, and T3, the failures rates for RMGIC were 8.0 %, 3.5 %, and 2.3 %, respectively, while the rates for RMD were 5.8 %, 1.2 %, and 2.3 %, respectively (Table 2). No statistically significant differences were observed in the debonding rates between the two groups (*P* > 0.05).

**Table 2:** Bracket failures of both materials at indicated time points (n = 87).

Time point	Number (rate) of bracket failures		χ2	*p* value
	RMGIC (n = 87)	RMD (n = 87)		
T1	7(8.0 %)	5(5.8 %)	0.090	0.765
T2	3(3.5 %)	1(1.2 %)	0.256	0.621
T3	2(2.3 %)	2(2.3 %)	<0.001	0.999

### 3.4 Impact of RMD on microbial community during orthodontic treatment

To explore the relationship between supragingival plaque biofilm composition during orthodontic treatment and the antimicrobial effects of RMD, metagenomic sequencing was performed on samples collected at T0 and T3. A total of 1,459,853,322 reads were obtained after filtering low-quality reads from 116 dental plaque samples, with an average of 50,339,770 clean reads per sample. A total of 5007 operational taxonomic units (OTUs) were identified, including 208 shared OTUs between the two groups.

**Fig. 5:**
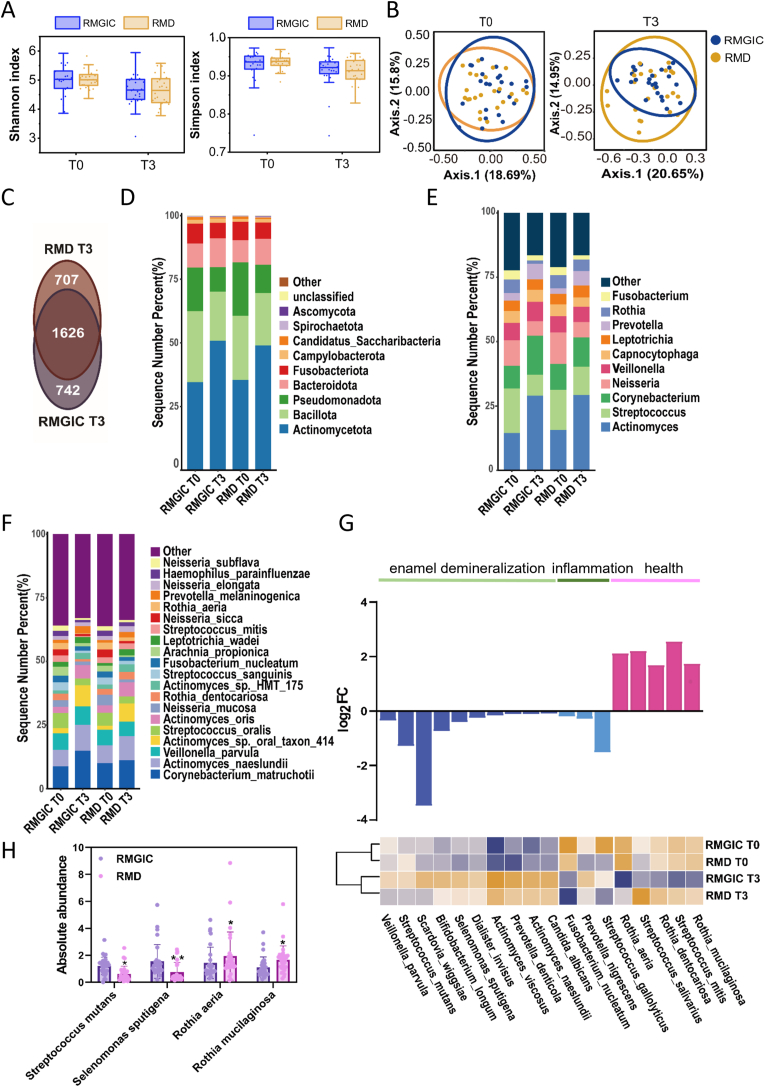
Impact of RMD on microbial community during orthodontic treatment. (**A**) Alpha diversity analysis of RMGIC and RMD groups at T0 and T3, based on the Shannon and Simpson indices. (**B**) Beta diversity analysis of RMGIC and RMD groups at T0 and T3, calculated using the weighted UniFrac distance. (**C**) Venn diagram showing the number of shared and unique bacterial species between RMGIC and RMD groups at T3. (**D**) Relative abundance of the top 10 phyla, (**E**) genera, and (**F**) the top 20 species of RMGIC and RMD groups at T0 and T3. (**G**) Relative abundance of species associated with enamel demineralization, inflammation, and oral health in the RMGIC and RMD groups. (**H**) Absolute abundance of pathogenic and beneficial species in the RMGIC and RMD groups. N = 25. All the results are presented as the mean ± SD. Statistical significance was determined using student *t*-test. ∗*P* < 0.05, ∗∗*P* < 0.01, versus the RMGIC group.

The α-diversity of the oral microbiome at T0 and T3 was assessed using the Shannon and Simpson indices, which evaluate species richness and evenness. Over the six-month period, no significant changes in microbial diversity were observed in either group (Fig. 5A). Furthermore, β-diversity comparison using weighted UniFrac distances at each time point revealed that the microbial populations in the RMD group were closely aligned with those in the RMGIC group (Fig. 5B). At the species level, the RMGIC and RMD groups shared 1626 common species at T3, with 742 unique species identified in the RMGIC group and 707 in the RMD group (Fig. 5C).

Next, the supragingival microbial communities between the RMGIC and RMD groups were compared at each time point, identifying 62 phyla, 1320 genera, and 978 species. While the overall microbial composition was similar between the two groups, certain core members of the oral microbiome exhibited slight but distinct shifts. Fig. 4, Fig. 5F presents the top 10 phyla, genera, and 20 species with the highest relative abundance in both groups at T0 and T3. At the phylum level, *Actinomycetota*, *Bacillota*, *Pseudomonadota*, *Bacteroidota*, and *Fusobacteriota* were dominant across all time points (Fig. 5D). At the genus level, compared to T0, the RMGIC group showed an increase in the relative abundance of *Prevotella* spp. and *Veillonella* spp. at T3, while the RMD group showed an increase in the relative abundance of *Streptococcus* spp. and *Rothia* spp. (Fig. 5E). At the species level, compared to T0, the RMGIC group showed an increase in the relative abundance of *Veillonella parvula* and *Prevotella melaninogenica*, while the RMD group showed an increase in the relative abundance of *R. aeria* and *Rothia dentocariosa* (Fig. 5F).

Furthermore, the relative abundance of species associated with enamel demineralization, periodontal inflammation, and oral health was analyzed between the two groups. Fig. 5G shows a bar plot depicting species with reduced (blue bars) or increased (pink bars) relative abundance in the RMD group compared to the RMGIC group. In conjunction with the heatmap, species associated with enamel demineralization and inflammation (represented by dark blue and light blue bars) exhibited increased relative abundance in the RMGIC group but decreased in the RMD group. Conversely, species linked to oral health (represented by pink bars) exhibited an opposite trend, with an increase in the RMD group.

RT-qPCR was performed to quantify the levels of common pathogenic bacteria, specifically *S*. *mutans* and *S. sputigena*, as well as common beneficial bacteria, namely *R. aeria* and *R. mucilaginosa*. RT-PCR analysis revealed that at T3, the levels of *S. mutans* and *S. sputigena* were significantly lower in the RMD group compared to the RMGIC group, whereas the levels of *R. aeria* and *R. mucilaginosa* were significantly higher in the RMD group (*P* < 0.05) (Fig. 5H).

### 3.5 Impact of RMD on microbial function during orthodontic treatment

**Fig. 6:**
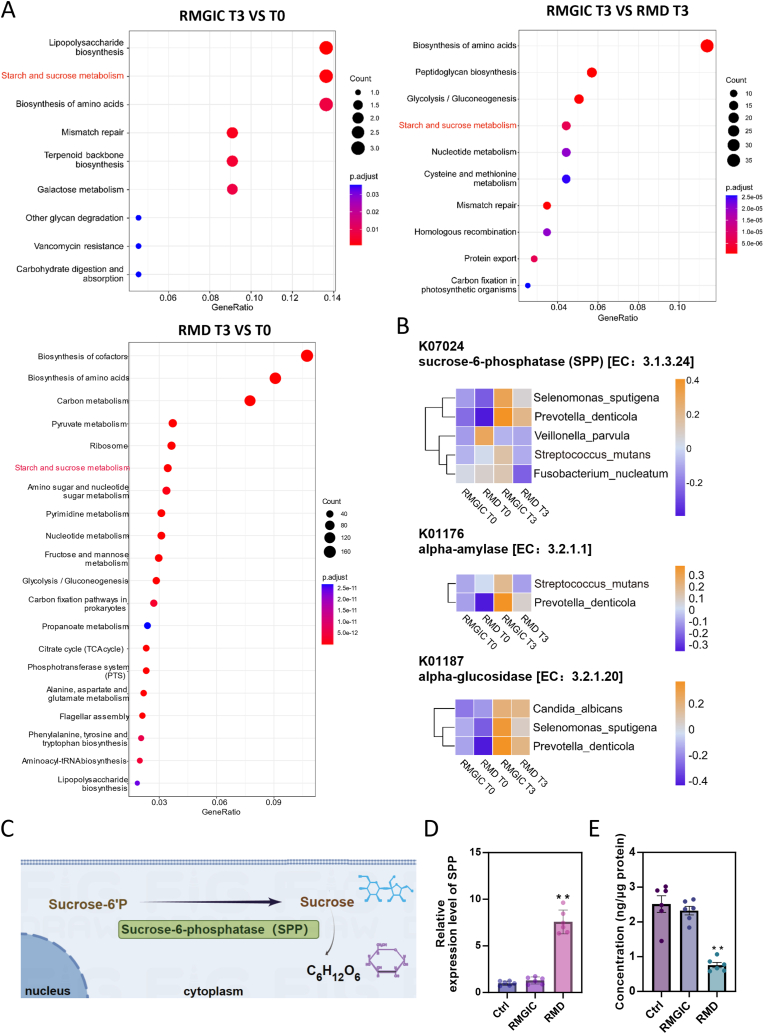
Impact of RMD on microbial function during orthodontic treatment. (**A**) KEGG analysis of the RMD and RMGIC groups during orthodontic treatment highlighted pathways with significant differences. (**B**) Three characteristic genes enriched in the starch and sucrose metabolism pathway and their species origins in the RMGIC and RMD groups during orthodontic treatment. (**C**) Sucrose-6-phosphatase (SPP) catalyzes the conversion of Sucrose-6′-phosphate (S-6′P) into sucrose. (**D**) RT-qPCR validation of SPP RNA expression levels after co-culture of RMGIC and RMD with *S. mutans*. N = 5. ∗∗*P* < 0.01, versus the RMGIC group. (**E**) The sucrose concentration after co-culture of RMGIC and RMD with *S. mutans*. N = 5. ∗∗*P* < 0.01, versus the RMGIC group. All the results are presented as the mean ± SD. Statistical significance was determined using ANOVA followed by Tukey's multiple comparison test.

To investigate the functional characteristics of the microbial communities between the RMGIC and RMD groups, gene annotation of the oral microbiome was performed using the Kyoto Encyclopedia of Genes and Genomes (KEGG) database. Comparative analysis revealed significant differences in metabolic pathways between T3 and T0 in the RMGIC group, including those related to lipopolysaccharide biosynthesis, starch and sucrose metabolism, and amino acid biosynthesis. In the RMD group, notable differences were observed in the pathways of cofactor biosynthesis, starch and sucrose metabolism, and amino acid biosynthesis between T3 and T0. Further comparison between the RMGIC and RMD groups at T3 highlighted significant differences in pathways involved in amino acid biosynthesis, peptidoglycan biosynthesis, and starch and sucrose metabolism. Focusing on shared differential pathways between RMGIC T3/T0, RMD T3/T0, and RMD T3/RMGIC T3, which may represent potential antimicrobial mechanisms of RMD, a significantly altered pathway common to all comparisons was identified: starch and sucrose metabolism (RMGIC T3 vs. T0, *P*.adj <0.001; RMD T3 vs. T0, *P*.adj <0.001; RMGIC T3 vs. RMD T3, *P*.adj <0.001) (Fig. 6A).

Fig. 6B highlights three characteristic genes enriched in the starch and sucrose metabolism pathway between the RMGIC and RMD groups: sucrose-6-phosphatase (SPP, EC:3.1.3.24, K07024), alpha-amylase (EC:3.2.1.1, K01176), and alpha-glucosidase (EC:3.2.1.20, K01187), with SPP showing the most pronounced changes. SPP catalyzes the conversion of Sucrose-6′-phosphate (S-6′P) into sucrose, as shown in Fig. 6C. Further analysis revealed that in the RMGIC group, cariogenic bacteria such as *S. mutans*, *S. sputigena*, *Prevotella denticola*, *Veillonella parvula*, *Fusobacterium nucleatum*, and *Candida albicans* showed an increase in sugar metabolism-related genes at T3 compared to T0. In contrast, these genes exhibited no significant changes in the RMD group between T0 and T3 (Fig. 6B).

Given the key role of *S. mutans* in dental caries, the expression levels of SPP RNA in *S. mutans* after co-culture with RMGIC and RMD were evaluated using RT-qPCR, further validating these findings. The results indicated that SPP expression was significantly elevated in the RMD group compared to the RMGIC group (*P* < 0.01), as shown in Fig. 6D. Correspondingly, following co-culture of *S. mutans* with the two materials in a sucrose-free medium, the sucrose concentration in the RMGIC group showed a moderate reduction compared to the control, without statistically significant differences. In contrast, the RMD group exhibited a significantly lower sucrose concentration than the RMGIC group (*P* < 0.01), as shown in Fig. 6E.

### 3.6 Impact of RMD on carbohydrate metabolism of microbial communities

**Fig. 7:**
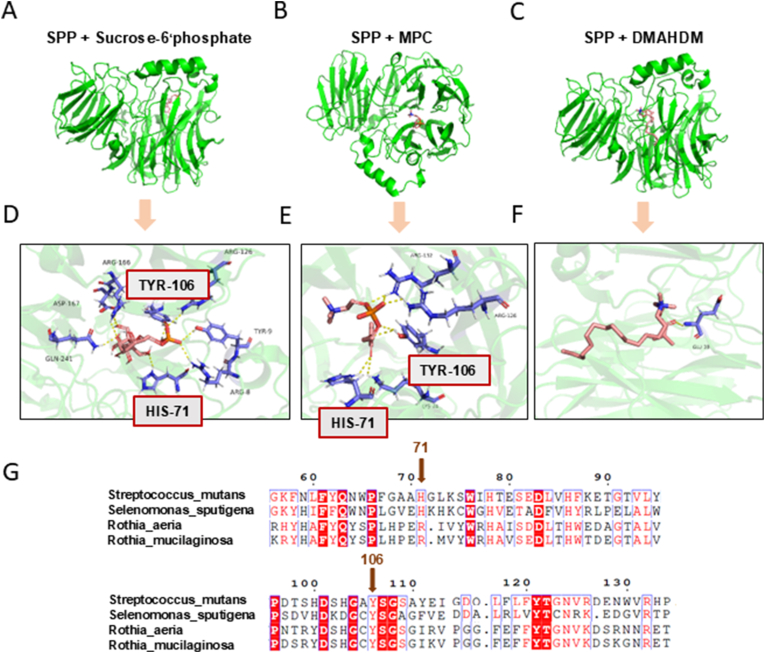
Molecular docking analysis. (A), **(B)**, and **(C)** show the binding modes and interaction sites of S-6′P, DMAHDM, and MPC with SPP. **(D)** Structural binding mode of S-6‘P to SPP. **(E)** Structural binding mode of MPC to SPP. **(F)** Structural binding mode of DMAHDM to SPP**. (G)** Sequence alignment showing the binding sites at positions 71 and 106 on SPP in *S. mutans*, *S. sputigena*, *R. aeria*, and *R. mucilaginosa*.

Molecular docking analysis revealed the binding modes and regulatory mechanisms of RMD on SPP in carbohydrate metabolism at the molecular level. Using AutoDock, the binding modes and interaction sites of MPC, DMAHDM, and S-6′P with SPP were examined to gain insight into their molecular interactions (Fig. 7A–C). As shown in Fig. 7D, S-6′P binds to SPP at multiple sites, including HIS-71, TYR-106, ARG-8, ARG-166, ASP-167, TYR-9, and GLN-241. Competitive binding between MPC and S-6′P at SPP was observed, with shared binding sites at HIS-71, and TYR-106 (Fig. 7E). In contrast, DMAHDM did not share any binding sites with SPP (Fig. 7F). Furthermore, the analysis revealed that the binding sites at HIS-71 and TYR-106 on SPP in *S. mutans* and *S. sputigena* correspond to the shared binding sites for MPC and S-6′P. However, in health-associated bacteria such as *R. aeria* and *R. mucilaginosa*, the residue at position 71 is arginine (Arg-71), differing from the corresponding site in pathogenic *S. mutans* and *S. sputigena* (Fig. 7G).

**Fig. 8:**
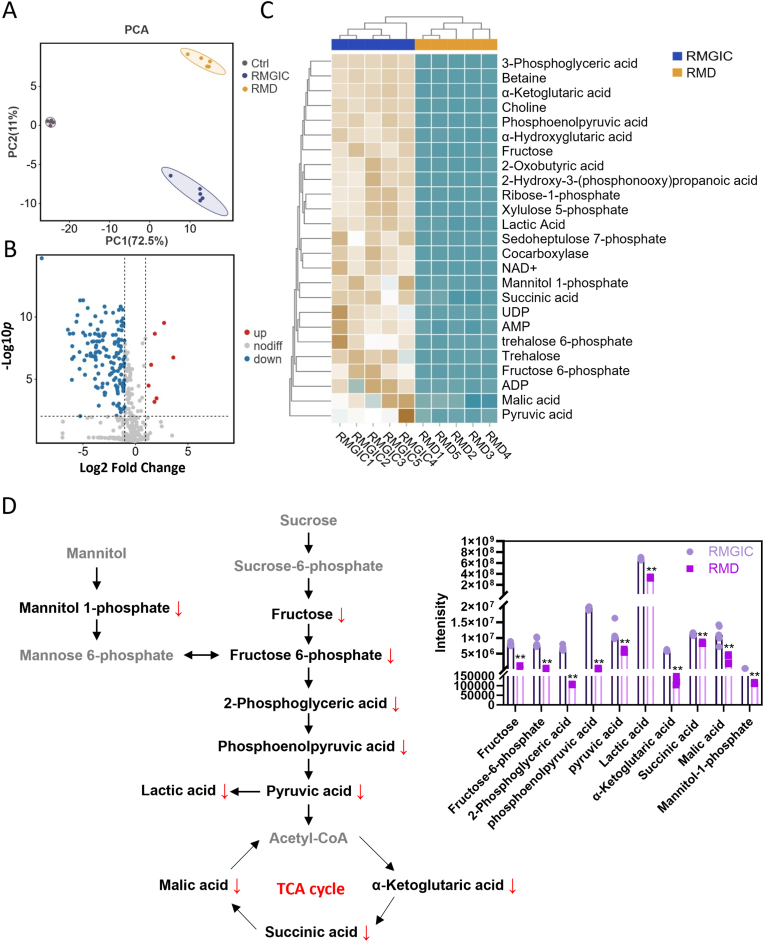
Metabolomic analysis of *S. mutans* co-cultured with RMGIC and RMD. (**A**) PCoA analysis of metabolites associated with carbohydrate metabolism. (**B**) Volcano plot of differential metabolites (DEMs). (**C**) Abundance analysis of 25 selected metabolites related to carbohydrate metabolism. (**D**) Intracellular levels of glycolysis and TCA cycle metabolites measured using liquid chromatography-tandem mass spectrometry. N = 5. All the results are presented as the mean ± SD. Statistical significance was determined using student *t*-test. ∗∗*P* < 0.01, versus the RMGIC group.

An untargeted metabolomic analysis was further conducted on *S. mutans* co-cultured with RMGIC and RMD, with a focus on the impact on carbohydrate metabolism. Polar metabolites were analyzed using flow injection time-of-flight mass spectrometry, following established protocols. Supervised discriminant analysis (PCA) revealed significant metabolic differences between the RMGIC and RMD groups (Fig. 8A). A total of 454 metabolites were identified, and based on the screening criteria of *P*. adj <0.01 with an absolute log2FC ≥ 1.155 differential metabolites (DEMs) were found, including 7 upregulated and 148 downregulated DEMs (Fig. 8B). Subsequently, 25 metabolites related to carbohydrate metabolism were chosen for abundance analysis. The results demonstrated consistently lower levels of these metabolites in the RMD group, indicating a pronounced inhibitory effect of RMD on carbohydrate metabolism (Fig. 8C). Key metabolites involved in glycolysis and the TCA cycle, such as fructose, fructose 6-phosphate, 2-phosphoglyceric acid, phosphoenolpyruvic acid, pyruvic acid, lactic acid, α-ketoglutaric acid, malic acid, succinic acid, and mannitol 1-phosphate, were significantly reduced in the RMD group (*P* < 0.01) (Fig. 8D). This reduction aligns with the observed decrease in sucrose levels in *S. mutans* following RMD treatment, confirming the substantial impact of RMD on the entire carbohydrate metabolic pathway of *S. mutans*.

## 4 Discussion

In recent years, research investigating the impact of changes in the oral microbiome on overall health has significantly increased. The oral microbiome, residing in the body's primary digestive organ, the oral cavity, metabolizes various dietary components, producing metabolites that may contribute to localized issues such as enamel demineralization, dental caries, and periodontitis. Additionally, it may lead to more complex systemic effects, including disruptions in the oral-cardiac axis [1], oral-brain axis [2], and oral-gut axis [3]. Given this situation, the inevitable changes in the oral microbiome during orthodontic treatment have garnered increasing attention, highlighting the need for sustained antimicrobial effects throughout the treatment.

With advancements in science and technology, antibacterial properties and anti-demineralization capabilities of orthodontic adhesives have been continuously optimized and modified. Common orthodontic adhesives include cements and resin. Among dental cements, resin-modified glass ionomer cement (RMGIC) is one of the most commonly used adhesives for orthodontic bracket bonding. RMGIC is a fluoride-releasing adhesive formed by blending traditional glass ionomer with resin monomers. However, the fluoride ion concentration released from RMGIC gradually decreases over time [43]. Additionally, the cured surface of RMGIC exhibits porosity and high roughness, facilitating bacterial adhesion and plaque accumulation [44,45]. Consequently, its efficacy in preventing enamel demineralization remains unclear [46]. Resin-based adhesives are polymer composites composed of inorganic fillers, resin matrices, and additives [47]. These materials typically lack fluoride release properties. Even with fluoride additives, effective long-term fluoride release is rarely achieved, limiting their ability to prevent enamel demineralization. Furthermore, various effective antimicrobial components can be incorporated into cements or resin adhesive. Based on composition, antimicrobial additives can be classified into: organic antimicrobial agents [48], inorganic antimicrobial agents [49], natural antimicrobial agents [50], and composite antimicrobial materials [26]. Previous studies have incorporated two antimicrobial agents, DMAHDM and MPC, into RMGIC, confirming their antibacterial properties [5]. This study further investigates the ordered assembly of DMAHDM and MPC and explores their selective antimicrobial mechanisms.

In the study of Yu et al., DMAHDM was incorporated into RMGIC at 0 %, 0.75 %, 1.5 %, 2.25 %, 3 %, 3.75 % and 4.5 % by mass. Increasing DMAHDM content reduced shear bond strength (SBS). At 3.75 %, SBS dropped below 8 MP, failing clinical requirements. In the remaining groups, the 3 % DMAHDM group showed maximal antibacterial effect, suppressing cariogenic bacteria and shifting the biofilm balance toward a non-cariogenic state [22]. In another study, MPC was incorporated into RMGI at 0 %, 1.5 %, 3 %, and 5 % by mass. RMGI containing 3 % MPC showed no significant SBS difference after 30 days of water-aging (*P* > 0.1). Meanwhile, the 3 % MPC group significantly reduced bacterial adhesion, lactic acid production, and colony-forming units of biofilms [28]. Ma et al. [5] demonstrated that RMGIC with 3 % MPC and 3 % DMAHDM maintained bonding strength after 6-month water-aging. And it has been proved that composite with 3 % DMAHDM +3 % MPC achieved greatest reduction in periodontal biofilm [51]. Therefore, this study combined 3 % DMAHDM and 3 % MPC. DMAHDM and MPC were combined to produce a freeze-dried DMAHDM@MPC powder. The powder was then incorporated into the RMGIC powder at 6 % by mass and thoroughly mixed.

The ordered assembly of different antimicrobial agents is key to enhancing the stability and efficacy of materials [52,53], especially during prolonged orthodontic treatment. Studies have shown that the ordered assembly and aggregation of antimicrobial materials such as antimicrobial peptides are vital for their antimicrobial activity and stability [54]. The findings of this study further confirm that DMAHDM and MPC form stable composite structures through non-covalent interactions, including electrostatic, hydrophobic, and hydrogen bonding interactions, thereby significantly enhancing the antimicrobial efficacy and stability of the material. TEM and XPS analyses further revealed that DMAHDM molecules are orderly encapsulated around the MPC core, forming a multilayered core-shell structure [55]. Collectively, these findings indicate that DMAHDM@MPC can self-assemble, given the long carbon chain of DMAHDM, which allows it to penetrate bacterial membranes [21], the outer DMAHDM nanoparticles of the DMAHDM@MPC structure penetrate bacterial membranes, while the core MPC continues to exert its antimicrobial effects within the bacteria, without concern for being adsorbed onto the bacterial surface. This ordered assembly preserves the unique properties of each component and enhances the composite material's stability and antimicrobial activity through synergy, providing an effective strategy for designing advanced antimicrobial materials.

Clinical trial results demonstrated that the RMD group consistently exhibited lower EDI and DD values compared to the RMGIC group across all evaluation periods. Both groups showed average EDI scores below Grade I (indicating mild enamel demineralization) and DD values under 12, suggesting relatively mild early-stage demineralization. These findings not only confirm the significant inhibitory effect of RMD on enamel demineralization during orthodontic treatment but also highlight the high sensitivity of the DD method in detecting material-specific differences in demineralization prevention. Compared to the study by Tufekci et al. [56], where pronounced white spot lesions emerged as early as six months into orthodontic treatment, both materials in this study demonstrated superior efficacy in preventing enamel demineralization.

Additionally, periodontal health remains a critical challenge during fixed orthodontic treatment. Previous studies reported significant increases in the plaque index, gingival index, and pocket depth [57]. Similarly, our study observed a progressive rise in periodontal health-related indices throughout the treatment period. While no intergroup differences in PI scores were noted at T1 and T2, the RMD group displayed significantly lower PI scores than the RMGIC group at T3. This indicates that RMD effectively inhibits plaque accumulation during fixed orthodontic treatment compared to RMGIC.

The combined results underscore the dual benefits of RMD: mitigating enamel demineralization and controlling plaque accumulation, thereby alleviating two major challenges in fixed orthodontic therapy.

During orthodontic treatment, maintaining the complexity and dynamic balance of the oral microbiome is crucial. Therefore, selectively suppressing harmful bacteria while preserving beneficial bacteria becomes a key issue in the antimicrobial process. This study used metagenomic techniques to examine the effects of RMGIC and RMD on the oral microbiome and shed light on their antimicrobial mechanisms. Results showed that after six months of treatment, no significant differences were observed in alpha and beta diversity between the two materials, indicating that neither material notably altered the overall microbiome structure, thus preserving the oral microbial balance. However, species-level analysis revealed subtle differences in core microbiota between the two groups. RMGIC promoted the growth of *Prevotella* and *Veillonella* spp. in the dental plaque biofilm, which are typical disease-associated bacteria [58,59], suggesting a potential shift toward biofilm maturation and the progression of carious lesions. In contrast, RMD maintained the stability of the abundance of *Streptococcus* and *Rothia* spp., which are typical health-associated bacteria [59,60], suggesting that RMD helps prevent the proliferation of potential pathogens. This distinction may reflect the different mechanisms by which these materials regulate microbiome balance. Moreover, species previously associated with demineralization and inflammation [16,61,62] were significantly more abundant in the RMGIC group compared to the RMD group. Notably, *S*. *mutans* is not only the primary pathogen responsible for enamel demineralization [63] but also a known contributor to infective endocarditis [64], chronic kidney disease [65], and atherosclerosis [66]. Furthermore, *F. nucleatum* and *Porphyromonas gingivalis* are linked to periodontal inflammation [16] and diabetes [67], with *Porphyromonas gingivalis* also associated with rheumatoid arthritis [68]. These findings suggest that RMGIC may elevate the risk of systemic diseases. Moreover, the abundance of health-associated species was reduced in the RMGIC group, while the RMD group maintained a stable level of beneficial microbial communities [69,70].This highlights the potential of RMD to regulate the oral microbiome during orthodontic treatment, which is crucial for mitigating the risks of enamel demineralization, periodontitis, and systemic diseases.

Previous studies on the antimicrobial mechanisms of DMAHDM and MPC have primarily focused on their physical properties, such as membrane insertion and inhibition of protein and bacterial adhesion [21,24,25]. To further investigate the antimicrobial targets of DMAHDM@MPC, particularly to elucidate its differential antimicrobial mechanisms, additional multi-omics research is necessary. Metagenomic functional analysis revealed significant differences in starch and sucrose metabolism pathways between the RMGIC and RMD groups during orthodontic treatment, providing key insights into the antimicrobial mechanisms of RMD. In the RMGIC group, from T0 to T3, the expression of genes involved in starch and sucrose metabolism pathways, such as SPP, alpha-amylase, and alpha-glucosidase, increased in pathogenic bacteria. In contrast, in the RMD group, the expression of these genes was suppressed in pathogenic bacteria. Specifically, SPP catalyzes the conversion of S-6′P into sucrose [71], alpha-amylase helps break down starch into maltose and dextrin [72], and alpha-glucosidase aids in the hydrolysis of disaccharides and some oligosaccharides into glucose and other simple sugars [73]. These enzymes play crucial roles in carbohydrate catabolism, facilitating the conversion of complex sugars into simpler forms, thereby supplying cariogenic bacteria with essential nutrients and increasing the risk of demineralization and caries. This suggests that RMD may help inhibit cariogenic activity by regulating the carbohydrate metabolism of pathogenic bacteria, thereby reducing the risk of dental decay during orthodontic treatment. Moreover, sucrose exhibits a highly cariogenic potential in the progression of dental caries [74], with SPP serving as a key enzyme that catalyzes the conversion of S-6′P into sucrose [71]. Since *S. mutans*, the primary cariogenic pathogen, plays a crucial role in the progression of enamel demineralization [63], the functional expression of SPP and sucrose production in *S. mutans* was investigated under the influence of RMGIC and RMD. Results showed that SPP RNA expression levels in *S. mutans* were higher in the RMD group compared to the RMGIC group, while the sucrose concentrations were significantly lower. This phenomenon may be attributed to the effect of RMD, which reduces sucrose levels, prompting *S. mutans* to upregulate SPP RNA expression in an attempt to synthesize more sucrose. These findings suggested that RMD may exert its antimicrobial effects by modulating sucrose metabolism in *S. mutans*, thereby effectively inhibiting bacterial growth and activity in high-sugar environments.

Furthermore, the underlying mechanisms were explored through molecular docking and metabolomic analysis. The molecular docking results confirmed that MPC can compete with S-6′P and bind to the SPP site, while DMAHDM does not directly interact with SPP, suggesting that DMAHDM exerts its antimicrobial effects through other mechanisms. This competitive binding likely impairs the effective binding of S-6′P to SPP, thereby reducing the efficiency of sucrose metabolism. Moreover, amino acid sequence alignment revealed that cariogenic species *S. mutans* and *S. sputigena* [63,75] exhibit higher affinity for MPC at the HIS-71 position. MPC preventing SPP from binding to S-6′P. In contrast, the health-associated species *R*. *aeria* and *R. mucilaginosa* [59,60] do not bind MPC at the Arg-71 position, thus minimizing the impact of MPC on their metabolic activity. These results highlight the competitive interaction between MPC and S-6′P, as well as the differences in binding affinities between pathogenic and health-associated bacteria. This mechanism may explain how RMD selectively inhibits cariogenic bacteria while exerting minimal effects on health-associated bacteria. Metabolomic analysis further demonstrated that RMD inhibited the entire glycolysis and TCA cycle pathways in *S. mutans*, significantly reducing the levels of key metabolites, particularly organic acids such as lactic acid. Given that acetic acid and lactic acid are known to contribute to enamel demineralization, other organic acids may also exhibit similar synergistic effects [76,77]. These results provide valuable insights into how RMD selectively inhibits sucrose metabolism and downstream processes in cariogenic bacteria, and helps maintain oral microbial balance during orthodontic treatment, effectively reducing enamel demineralization and the risk of caries.

However, this study still has certain limitations. First, the sample size was relatively limited. Expanding the cohort size and extending follow-up durations in future research would enhance result precision. Second, the study only explored carbohydrate metabolism. To gain a comprehensive understanding of the material's antibacterial properties, it is essential to investigate other metabolic pathways. Finally, further theoretical calculations and experiments are needed to optimize the component ratio for better antibacterial performance.

## 5 Conclusion

MPC and DMAHDM were assembled into nanospheres, termed DMAHDM@MPC, which were subsequently mixed with RMGIC to form RMD that effectively inhibits cariogenic bacteria. The mechanism involves RMD selectively targeting cariogenic bacteria by competitively binding to the SPP, disrupting its interaction with S-6′P. This process blocks carbohydrate metabolism in cariogenic bacteria, significantly reducing sucrose utilization and acid production, thereby preventing enamel demineralization and caries. These properties make RMD an ideal material for protecting oral health during orthodontic treatment.

## CRediT authorship contribution statement

**Chengjun Su:** Writing – review & editing, Writing – original draft, Methodology, Conceptualization. **Mengyao Zhu:** Writing – review & editing, Methodology, Conceptualization. **Yiman Guo:** Writing – review & editing, Methodology, Conceptualization. **Jiachen Sun:** Writing – review & editing, Methodology, Conceptualization. **Miao Liu:** Visualization. **Yansong Ma:** Investigation. **Yan Xu:** Investigation. **Yuxing Bai:** Supervision. **Xiaoxia Che:** Supervision. **Ning Zhang:** Writing – review & editing, Supervision.

## Funding statement

This work was supported by China University Research Innovation Fund-Infection and Control Special Project (2024GR057), 10.13039/501100001809National Natural Science Foundation of China (82370989) and Innovation Research Team Project of Beijing Stomatological Hospital, 10.13039/501100002799Capital Medical University (CXTD202203).

## Declaration of competing interest

The authors declare that they have no known competing financial interests or personal relationships that could have appeared to influence the work reported in this paper.

## Data Availability

Data will be made available on request.

## References

[1] Li Y., Zhu M., Liu Y., Luo B., Cui J., Huang L., Chen K., Liu Y. (2022). The oral microbiota and cardiometabolic health: a comprehensive review and emerging insights. Front. Immunol..

[2] Maitre Y., Micheneau P., Delpierre A., Mahalli R., Guerin M., Amador G., Denis F. (2020). Did the brain and oral microbiota talk to each other? A review of the literature. J. Clin. Med..

[3] Yin W., Ludvigsson J.F., Liu Z., Roosaar A., Axell T., Ye W. (2017). Inverse association between poor oral health and inflammatory bowel diseases. Clin. Gastroenterol. Hepatol..

[4] Inchingolo A.D., Malcangi G., Semjonova A., Inchingolo A.M., Patano A., Coloccia G., Ceci S., Marinelli G., Di Pede C., Ciocia A.M., Mancini A., Palmieri G., Barile G., Settanni V., De Leonardis N., Rapone B., Piras F., Viapiano F., Cardarelli F., Nucci L., Bordea I.R., Scarano A., Lorusso F., Palermo A., Costa S., Tartaglia G.M., Corriero A., Brienza N., Di Venere D., Inchingolo F., Dipalma G. (2022). Oralbiotica/oralbiotics: the impact of oral microbiota on dental health and demineralization: a systematic review of the literature. Children.

[5] Ma Y., Su C., Yang H., Xu H.H.K., Bai Y., Xu Y., Che X., Zhang N. (2023). Influence of resin modified glass ionomer cement incorporating protein-repellent and antimicrobial agents on supragingival microbiome around brackets: an in-vivo split-mouth 3-month study. PeerJ.

[6] Ogaard B., Rølla G., Arends J., ten Cate J.M. (1988). Orthodontic appliances and enamel demineralization. Part 2. Prevention and treatment of lesions. Am. J. Orthod. Dentofacial Orthop..

[7] Sukontapatipark W., el-Agroudi M.A., Selliseth N.J., Thunold K., Selvig K.A. (2001). Bacterial colonization associated with fixed orthodontic appliances. A scanning electron microscopy study. Eur. J. Orthod..

[8] Negrini T.C., Ren Z., Miao Y., Kim D., Simon-Soro A., Liu Y., Koo H., Arthur R.A. (2022). Dietary sugars modulate bacterial-fungal interactions in saliva and inter-kingdom biofilm formation on apatitic surface. Front. Cell. Infect. Microbiol..

[9] Schachtele D.F., Leung W.L. (1975). Effect of sugar analogues on growth, sugar utilization, and acid production by Streptococcus mutans. J. Dent. Res..

[10] Kado I., Hisatsune J., Tsuruda K., Tanimoto K., Sugai M. (2020). The impact of fixed orthodontic appliances on oral microbiome dynamics in Japanese patients. Sci. Rep..

[11] Ogaard B., Rolla G., Arends J. (1988). Orthodontic appliances and enamel demineralization. Part 1. Lesion development. Am. J. Orthod. Dentofacial Orthop..

[12] Flynn L.N., Julien K., Noureldin A., Buschang P.H. (2022). The efficacy of fluoride varnish vs a filled resin sealant for preventing white spot lesions during orthodontic treatment. Angle Orthod..

[13] Mehta A., Paramshivam G., Chugh V.K., Singh S., Halkai S., Kumar S. (2015). Effect of light-curable fluoride varnish on enamel demineralization adjacent to orthodontic brackets: an in-vivo study. Am. J. Orthod. Dentofacial Orthop..

[14] Marks L.A., Verbeeck R.M., De Maeyer E.A., Martens L.C. (2000). Effect of maturation on the fluoride release of resin-modified glass ionomer and polyacid-modified composite resin cements. Biomaterials.

[15] Guo Z., Richardson J.J., Kong B., Liang K. (2020). Nanobiohybrids: materials approaches for bioaugmentation. Sci. Adv..

[16] Wang Q., Liu S., Chen W., Ni Y., Zeng S., Chen P., Xu Y., Nie W., Zhou Y. (2024). Strong, bacteriostatic and transparent polylactic acid-based composites by incorporating quaternary ammonium cellulose nanocrystals. Int. J. Biol. Macromol..

[17] Davari A., Mosaddegh A., Daneshkazemi A., Frahat F., Hakimzadeh A., Abbasi S. (2022). Evaluation of shear bond strength and antimicrobial effects of resin-modified glass ionomer containing titanium oxide and silver nanoparticles. J. Dent. Sch..

[18] Shirazi M., Qazvini F.F., Mohamadrezaie S. (2023). Antimicrobial properties of glass-ionomer cement incorporated with zinc oxide nanoparticles against mutans streptococci and lactobacilli under orthodontic bands: an in vivo split-mouth study. Dent. Res. J. (Isfahan).

[19] Lin J., Zhu J., Gu X., Wen W., Li Q., Fischer-Brandies H., Wang H., Mehl C. (2011). Effects of incorporation of nano-fluorapatite or nano-fluorohydroxyapatite on a resin-modified glass ionomer cement, Acta. Biomaterials.

[20] Ramasamy M., Das M., An S.S., Yi D.K. (2014). Role of surface modification in zinc oxide nanoparticles and its toxicity assessment toward human dermal fibroblast cells. Int. J. Nanomed..

[21] Zhou Z., Zhou S., Zhang X., Zeng S., Xu Y., Nie W., Zhou Y., Xu T., Chen P. (2023). Quaternary ammonium salts: insights into synthesis and new directions in antibacterial applications. Bioconjug. Chem..

[22] Yu W., Ren C., Zhang N., Cao L., Weir M.D., Yang K., Xu H.H.K., Bai Y. (2023). Dual function of anti-biofilm and modulating biofilm equilibrium of orthodontic cement containing quaternary ammonium salt. Dent. Mater. J..

[23] Zhang N., Ma J., Melo M.A., Weir M.D., Bai Y., Xu H.H. (2015). Protein-repellent and antibacterial dental composite to inhibit biofilms and caries. J. Dent..

[24] Kim H.K., Park J.H., Jang M.J., Han S.J., Cho Y.S., Park H.H. (2024). Flexible and transparent nanohole-patterned films with antibacterial properties against Staphylococcus aureus. J. Mater. Chem. B.

[25] Song X., Man J., Qiu Y., Wang J., Li R., Zhang Y., Cui G., Li J., Li J., Chen Y. (2024). Study of hydration repulsion of zwitterionic polymer brushes resistant to protein adhesion through molecular simulations. ACS Appl. Mater. Interfaces.

[26] Ma Y., Zhang N., Weir M.D., Bai Y., Xu H.H.K. (2017). Novel multifunctional dental cement to prevent enamel demineralization near orthodontic brackets. J. Dent..

[27] Makvandi P., Jamaledin R., Jabbari M., Nikfarjam N., Borzacchiello A. (2018). Antibacterial quaternary ammonium compounds in dental materials: a systematic review. Dent. Mater..

[28] Zhang N., Zhang K., Melo M.A., Chen C., Fouad A.F., Bai Y., Xu H.H. (2016). Novel protein-repellent and biofilm-repellent orthodontic cement containing 2-methacryloyloxyethyl phosphorylcholine. J. Biomed. Mater. Res. B Appl. Biomater..

[29] Lesaffre E., Philstrom B., Needleman I., Worthington H. (2009). The design and analysis of split-mouth studies: what statisticians and clinicians should know. Stat. Med..

[30] Banks P.A., Richmond S. (1994). Enamel sealants: a clinical evaluation of their value during fixed appliance therapy. Eur. J. Orthod..

[31] Mizrahi E. (1982). Enamel demineralization following orthodontic treatment. Am. J. Orthod..

[32] Löe H. (1967). The gingival index, the plaque index and the retention index systems. J. Periodontol..

[33] Katz J., Yang Q.B., Zhang P., Potempa J., Travis J., Michalek S.M., Balkovetz D.F. (2002). Hydrolysis of epithelial junctional proteins by Porphyromonas gingivalis gingipains. Infect. Immun..

[34] Wood D.E., Lu J., Langmead B. (2019). Improved metagenomic analysis with Kraken 2. Genome Biol..

[35] Bolger A.M., Lohse M., Usadel B. (2014). Trimmomatic: a flexible trimmer for Illumina sequence data. Bioinformatics.

[36] Langmead B., Salzberg S.L. (2012). Fast gapped-read alignment with Bowtie 2. Nat. Methods.

[37] Lu J., Breitwieser F.P., Thielen P., Salzberg S.L. (2017). Bracken: estimating species abundance in metagenomics data. PeerJ Comput. Sci..

[38] Kanehisa M., Furumichi M., Tanabe M., Sato Y., Morishima K. (2017). KEGG: new perspectives on genomes, pathways, diseases and drugs. Nucleic Acids Res..

[39] Lukic D., Karygianni L., Flury M., Attin T., Thurnheer T. (2020). Endodontic-like oral biofilms as models for multispecies interactions in endodontic diseases. Microorganisms.

[40] Neves B.G., Stipp R.N., Bezerra D.D.S., Guedes S.F.F., Rodrigues L.K.A. (2018). Quantitative analysis of biofilm bacteria according to different stages of early childhood caries. Arch. Oral Biol..

[41] Tsuzukibashi O., Uchibori S., Shinozaki-Kuwahara N., Saito M., Kobayashi T., Fukumoto M. (2013). New primer design for identification of oral rothia, including R. Aeria, using multiplex PCR. Int. J. Oral-Med. Sci..

[42] Aljehani A., Yang L., Shi X.Q. (2007). In vitro quantification of smooth surface caries with DIAGNOdent and the DIAGNOdent pen. Acta Odontol. Scand..

[43] Rogers S., Chadwick B., Treasure E. (2010). Fluoride-containing orthodontic adhesives and decalcification in patients with fixed appliances: a systematic review. Am. J. Orthod. Dentofacial Orthop..

[44] Badawi H., Evans R.D., Wilson M., Ready D., Noar J.H., Pratten J. (2003). The effect of orthodontic bonding materials on dental plaque accumulation and composition in vitro. Biomaterials.

[45] Ahn S.J., Lim B.S., Lee S.J. (2010). Surface characteristics of orthodontic adhesives and effects on streptococcal adhesion. Am. J. Orthod. Dentofacial Orthop..

[46] Benson P.E., Alexander-Abt J., Cotter S., Dyer F.M.V., Fenesha F., Patel A., Campbell C., Crowley N., Millett D.T. (2019). Resin-modified glass ionomer cement vs composite for orthodontic bonding: a multicenter, single-blind, randomized controlled trial. Am. J. Orthod. Dentofacial Orthop..

[47] Masood T.M., Abbassy M.A., Bakry A.S., Matar N.Y., Hassan A.H. (2018). Fourier-transform infrared spectroscopy/attenuated total reflectance analysis for the degree of conversion and shear bond strength of Transbond XT adhesive system. Clin. Cosmet. Invest. Dent..

[48] Zhou H., Li F., Weir M.D., Xu H.H. (2013). Dental plaque microcosm response to bonding agents containing quaternary ammonium methacrylates with different chain lengths and charge densities. J. Dent..

[49] Liu Y., Zhang L., Niu L.N., Yu T., Xu H.H.K., Weir M.D., Oates T.W., Tay F.R., Chen J.H. (2018). Antibacterial and remineralizing orthodontic adhesive containing quaternary ammonium resin monomer and amorphous calcium phosphate nanoparticles. J. Dent..

[50] Hoglund K.B., Barnett B.K., Watson S.A., Melgarejo M.B., Kang Y. (2020). Activity of bioactive garlic compounds on the oral microbiome: a literature review. Gen. Dent..

[51] Wang L., Xie X., Imazato S., Weir M.D., Reynolds M.A., Xu H.H.K. (2016). A protein-repellent and antibacterial nanocomposite for Class-V restorations to inhibit periodontitis-related pathogens. Mater. Sci. Eng., C.

[52] Li J., Chen Z., Zhou M., Jing J., Li W., Wang Y., Wu L., Wang L., Wang Y., Lee M. (2016). Polyoxometalate-driven self-assembly of short peptides into multivalent nanofibers with enhanced antibacterial activity. Angew. Chem. Int. Ed. Engl..

[53] Liu S.Q., Venkataraman S., Ong Z.Y., Chan J.M., Yang C., Hedrick J.L., Yang Y.Y. (2014). Overcoming multidrug resistance in microbials using nanostructures self-assembled from cationic bent-core oligomers. Small (Weinh.).

[54] Malekkhaiat Häffner S., Malmsten M. (2018). Influence of self-assembly on the performance of antimicrobial peptides. Curr. Opin. Colloid Interface Sci..

[55] Li J., Hou Y., Wu H., Chen C., Fu X., Liu J., Li L., Shang S., Deng G. (2025). A poly (vinyl alcohol) coated core-shell nanoparticle with a tunable surface for pH and glutathione dual-responsive drug delivery. Colloids Surf., B Biointerfaces.

[56] Tufekci E., Dixon J.S., Gunsolley J.C., Lindauer S.J. (2011). Prevalence of white spot lesions during orthodontic treatment with fixed appliances. Angle Orthod..

[57] Kumar S., Kumar S., Hassan N., Anjan R., Shaikh S., Bhowmick D. (2021). A comparative assessment of the effect of professional oral hygiene measures on the periodontal health of patients undergoing fixed orthodontic appliance therapy. J. Pharm. BioAllied Sci..

[58] da Costa Rosa T., de Almeida Neves A., Azcarate-Peril M.A., Divaris K., Wu D., Cho H., Moss K., Paster B.J., Chen T., L B.F.-F., Fidalgo T.K.S., Tadeu Lopes R., Valente A.P., R R.A., de Aguiar Ribeiro A. (2021). The bacterial microbiome and metabolome in caries progression and arrest. J. Oral Microbiol..

[59] Rosier B.T., Takahashi N., Zaura E., Krom B.P., MartInez-Espinosa R.M., van Breda S.G.J., Marsh P.D., Mira A. (2022). The importance of nitrate reduction for oral health. J. Dent. Res..

[60] Sulyanto R.M., Thompson Z.A., Beall C.J., Leys E.J., Griffen A.L. (2019). The predominant oral microbiota is acquired early in an organized pattern. Sci. Rep..

[61] Baraniya D., Chen T., Nahar A., Alakwaa F., Hill J., Tellez M., Ismail A., Puri S., Al-Hebshi N.N. (2020). Supragingival mycobiome and inter-kingdom interactions in dental caries. J. Oral Microbiol..

[62] Tanner A.C., Sonis A.L., Lif Holgerson P., Starr J.R., Nunez Y., Kressirer C.A., Paster B.J., Johansson I. (2012). White-spot lesions and gingivitis microbiotas in orthodontic patients. J. Dent. Res..

[63] Seminario A., Broukal Z., Ivancakova R. (2005). Mutans streptococci and the development of dental plaque, Prague. Med. Repos..

[64] Lockwood W.R., Lawson L.A., Smith D.L., McNeil K.M., Morrison F.S. (1974). Streptococcus mutans endocarditis. Report of a case. Ann. Intern. Med..

[65] Menezes C.R., Pereira A.L., Ribeiro C.C., Chaves C.O., Guerra R.N., Thomaz E.B., Monteiro-Neto V., Alves C.M. (2019). Is there association between chronic kidney disease and dental caries? A case-controlled study. Med. Oral Patol. Oral Cir. Bucal.

[66] Kesavalu L., Lucas A.R., Verma R.K., Liu L., Dai E., Sampson E., Progulske-Fox A. (2012). Increased atherogenesis during Streptococcus mutans infection in ApoE-null mice. J. Dent. Res..

[67] Tervonen T., Oliver R.C., Wolff L.F., Bereuter J., Anderson L., Aeppli D.M. (1994). Prevalence of periodontal pathogens with varying metabolic control of diabetes mellitus. J. Clin. Periodontol..

[68] Courbon G., Rinaudo-Gaujous M., Blasco-Baque V., Auger I., Caire R., Mijola L., Vico L., Paul S., Marotte H. (2019). Porphyromonas gingivalis experimentally induces periodontis and an anti-CCP2-associated arthritis in the rat. Ann. Rheum. Dis..

[69] Colombo A.P., Boches S.K., Cotton S.L., Goodson J.M., Kent R., Haffajee A.D., Socransky S.S., Hasturk H., Van Dyke T.E., Dewhirst F., Paster B.J. (2009). Comparisons of subgingival microbial profiles of refractory periodontitis, severe periodontitis, and periodontal health using the human oral microbe identification microarray. J. Periodontol..

[70] Ihara Y., Takeshita T., Kageyama S., Matsumi R., Asakawa M., Shibata Y., Sugiura Y., Ishikawa K., Takahashi I., Yamashita Y. (2019). Identification of initial colonizing bacteria in dental plaques from young adults using full-length 16S rRNA gene sequencing. mSystems.

[71] Daude D., Remaud-Simeon M., Andre I. (2012). Sucrose analogs: an attractive (bio)source for glycodiversification. Nat. Prod. Rep..

[72] Paul J.S., Gupta N., Beliya E., Tiwari S., Jadhav S.K. (2021). Aspects and recent trends in microbial α-amylase: a review. Appl. Biochem. Biotechnol..

[73] Zhang X., Li G., Wu D., Yu Y., Hu N., Wang H., Li X., Wu Y. (2020). Emerging strategies for the activity assay and inhibitor screening of alpha-glucosidase. Food Funct..

[74] Cai J.N., Jung J.E., Lee M.H., Choi H.M., Jeon J.G. (2018). Sucrose challenges to Streptococcus mutans biofilms and the curve fitting for the biofilm changes. FEMS Microbiol. Ecol..

[75] Cho H., Ren Z., Divaris K., Roach J., Lin B.M., Liu C., Azcarate-Peril M.A., Simancas-Pallares M.A., Shrestha P., Orlenko A., Ginnis J., North K.E., Zandona A.G.F., Ribeiro A.A., Wu D., Koo H. (2023). Selenomonas sputigena acts as a pathobiont mediating spatial structure and biofilm virulence in early childhood caries. Nat. Commun..

[76] Detman A., Mielecki D., Chojnacka A., Salamon A., Blaszczyk M.K., Sikora A. (2019). Cell factories converting lactate and acetate to butyrate: Clostridium butyricum and microbial communities from dark fermentation bioreactors. Microb. Cell Fact..

[77] Dashper S.G., Reynolds E.C. (1996). Lactic acid excretion by Streptococcus mutans. Microbiology (Reading, England).

